# Pulmonary Artery Sarcoma: A Rare Entity

**DOI:** 10.1177/2324709614529416

**Published:** 2014-04-03

**Authors:** Maha Shomaf, Nathir Obeidat, Fatin Al-Fares, Saleh Najjar

**Affiliations:** 1 The University of Jordan, Department of Pathology; 2 The University of Jordan, Department of Internal Medicine; 3 Jordan University Hospital, The Department of Pathology

**Keywords:** pulmonary, sarcoma, thromboembolism

## Abstract

Pulmonary artery sarcomas (PAS) are extremely rare sarcomas of uncertain histogenesis that often mimic pulmonary thromboemboli. This is a report of a 60-year-old female patient who presented with recurrent chest pain and cough. The patient was first diagnosed with pulmonary embolism but she did not improve on anticoagulant therapy. Follow-up imaging studies revealed a mass in the left hilar region extending into the pulmonary trunk and branches of the left pulmonary artery. The tru-cut biopsy revealed an undifferentiated sarcoma. The patient died 10 months after her initial presentation.

## Introduction

Pulmonary artery sarcoma (PAS) is a rare entity. Its importance comes from the fact that it can be misdiagnosed as pulmonary thromboembolism, because they share common clinical presentation.

## The Case

**Figure 1.**
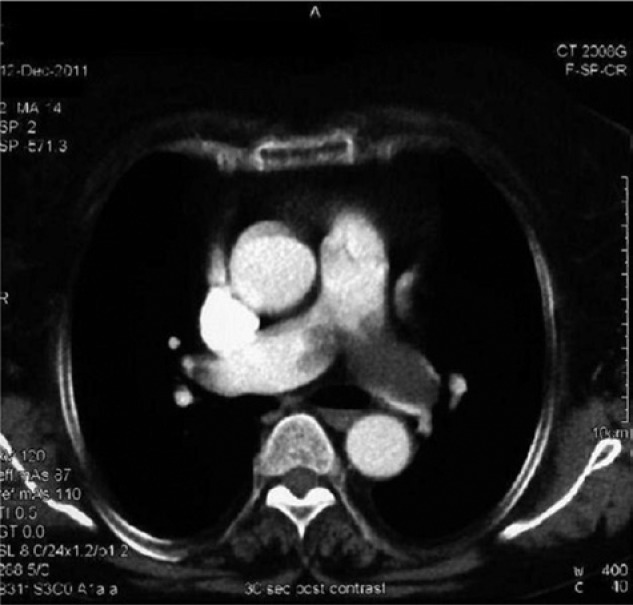
Computed tomography scan of chest showing saddle-shaped filling defect in the main pulmonary trunk extending into the left main pulmonary artery and its segmental branches, and no pulmonary nodules or pleural effusion were present.

A 60-year-old hypertensive, nonsmoker female patient presented to the emergency room with chest pain of 5 hours duration radiating to the left arm. On physical examination her blood pressure was 150/80 mm Hg, the pulse rate was 82 beats per minute, and the respiratory rate was 18 per minute. Her electrocardiogram was reported as normal. The echocardiogram revealed diastolic dysfunction and trace pulmonary regurgitation. The patient was discharged against medical advice. Four months later she presented with cough of one month duration. Pulmonary function test was done and showed an obstructive pattern. Computed tomography (CT) scan of chest revealed cardiomegaly and saddle-shaped filling defect in the main pulmonary trunk extending into the left main pulmonary artery and its segmental branches, and no pulmonary nodules or pleural effusion were present (Figure 1).

A chronic pulmonary thromboembolism was suspected, and thus the patient was admitted to the hospital although she was not hypoxic or distressed.

**Figure 2.**
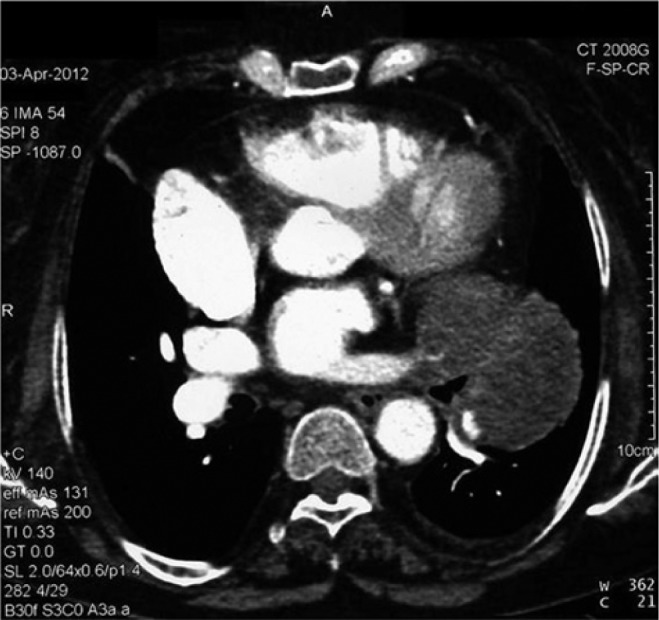
Chest spiral CT scan with contrast showing a large lobulated hypodense mass in the left hilar and perihilar region extending into pulmonary trunk, both pulmonary arteries, and branches of left pulmonary artery, which encases the left pulmonary veins it measures 10x12 cm.

**Figure 3.**
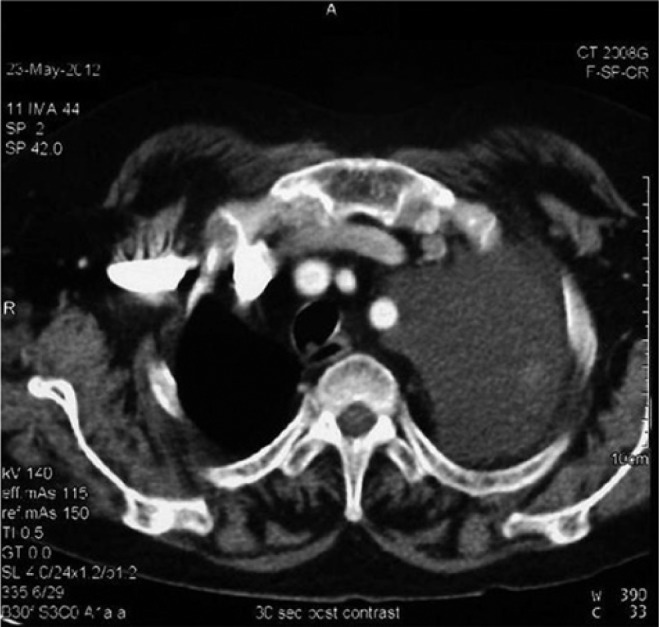
Chest CT scan with contrast showing a large mass involving the whole left lung with extension to the pulmonary trunk, with mediastinal shift to the right side.

**Figure 4.**
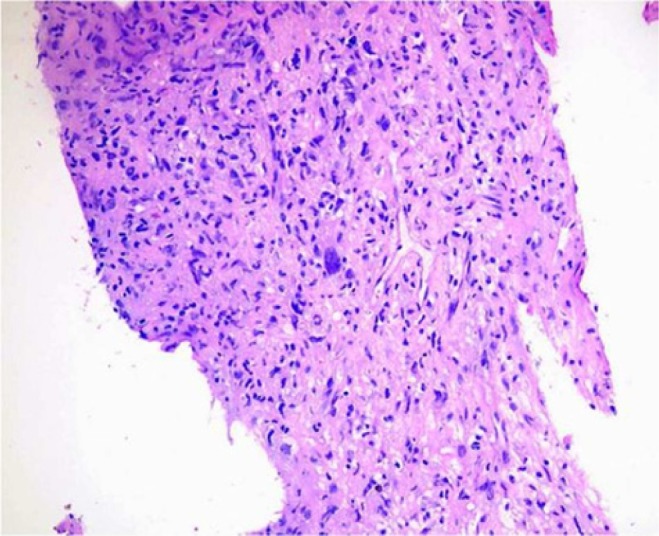
Hematoxylin–eosin stain showing a poorly differentiated sarcoma with marked cellular pleomorphism, 200× magnification.

**Figure 5.**
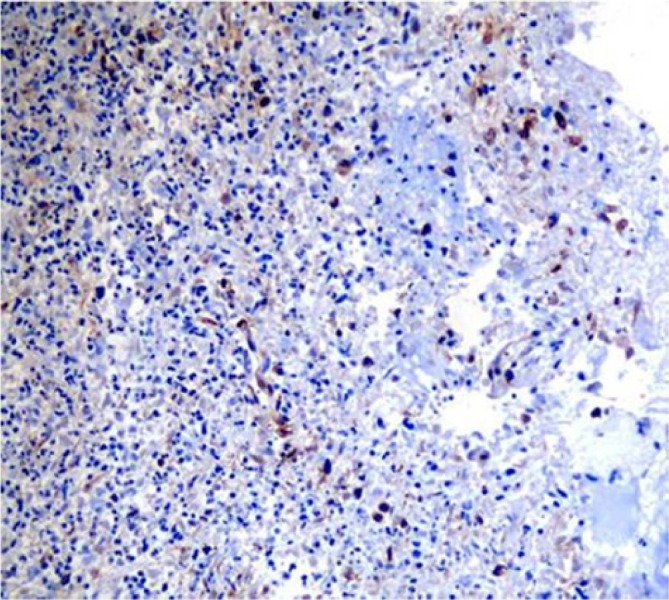
Positive vimentin immunohistochemical stain, 200× magnification.

**Figure 6.**
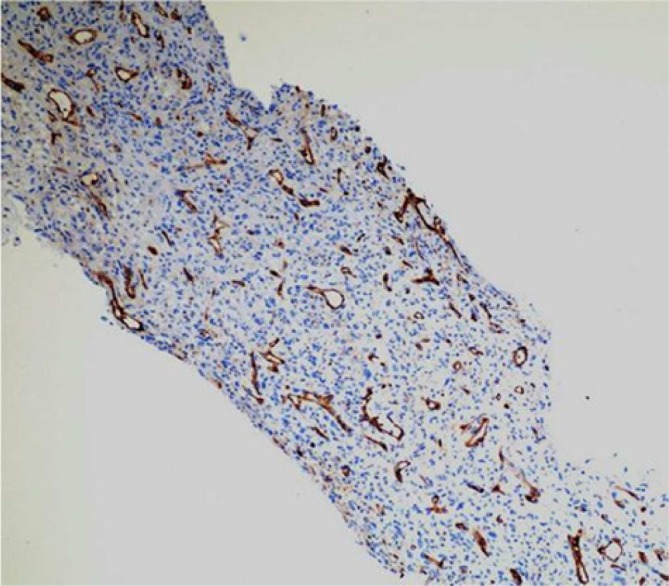
Negative CD31 immunohistochemical stain, 50× magnification.

**Figure 7.**
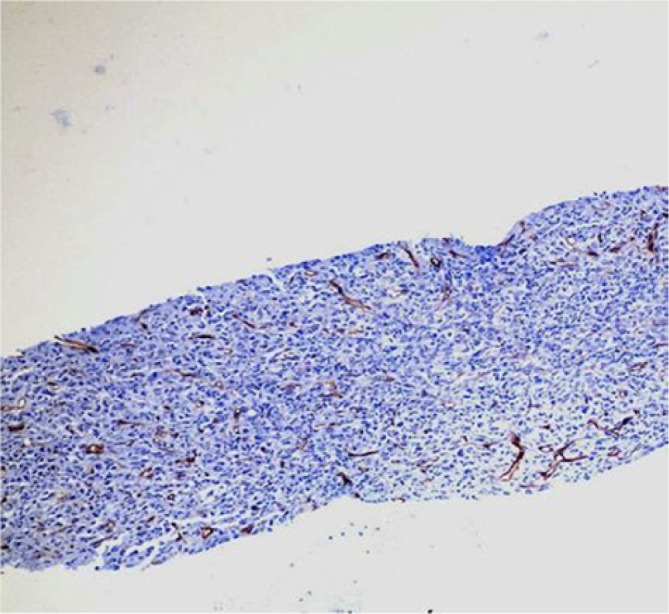
Negative CD34 immunohistochemical stain, 50× magnification.

A bilateral lower limb Doppler ultrasound proved to be negative for thrombosis. Thrombophilia workup revealed a homozygous mutation of A1298C MTHFR by polymerase chain reaction. The patient received anticoagulant therapy but showed no improvement. Four months later a chest (spiral) CT scan with contrast was done and showed a large lobulated hypodense mass in the left hilar and perihilar region extending into the pulmonary trunk, both pulmonary arteries, and branches of the left pulmonary artery (Figure 2). On this admission the echocardiogram revealed a diastolic dysfunction, pulmonary valve regurge, and pulmonary hypertension with pulmonary artery pressure of 42 mm Hg. A bronchoalveolar lavage was performed with an endobronchial biopsy, which was reported as superficial respiratory epithelium. On her follow-up visit, a chest CT scan showed a large mass involving the left lung with extension to the pulmonary trunk and mediastinal shift to the right, hilar lymph nodes enlargement, and moderate left pleural effusion were present (Figure 3). A CT-guided tru-cut biopsy was performed and the histopathology revealed a poorly differentiated sarcoma with extensive necrosis (Figure 4). The tumor cells show brisk mitotic activity including abnormal forms. Immunohistochemical stains were done and showed positivity for vimentin (Figure 5), but CK, LCA, CD31, CD34, actin, caldesmon, desmin, and β-catenin stains were negative (Figure 6 and 7). The diagnosis of undifferentiated pulmonary artery intimal sarcoma was made based on these findings.

The patient refused any medical intervention and was discharged against medical advice. Ten months later she presented with respiratory failure. Cardiopulmonary resuscitation was attempted but failed, and unfortunately the patient died.

## Discussion

Mandelstamm described the first case of pulmonary artery sarcoma in 1923.^1^ It is a very rare aggressive tumor with a median survival time of 1.5 months without surgical resection.^2^ Intimal sarcoma of the pulmonary artery is usually misdiagnosed as pulmonary thromboembolism due to similar clinical presentation and radiological findings. In our case, the saddle-shaped intraluminal filling defect in the main pulmonary trunk shown on the CT scan raised the suspicion of pulmonary embolism although the patient did not complain initially of hypoxia, tachycardia, or respiratory distress that would have supported the diagnosis of pulmonary embolism, not to mention the negative lower limbs Doppler ultrasound. PAS patients present usually with hemoptysis, weight loss, fever, and digital clubbing.^3^

Intimal sarcomas belong to tumors of uncertain differentiation in the World Health Organization classification of tumors of soft tissue and bone.^4^ In our case, the tumor on immunohistochemical grounds showed only positive vimentin staining, which indicates a mesenchymal origin; however, the endothelial markers CD31 and CD34 were negative in malignant cells excluding an endothelial derivation. Vasuri et al proposed the idea that these neoplasms might originate from a vessel wall-resident stem cell, such as the hemangioblast or an embryonic-like stem cell. Yet the possibility of the inverse process, that is, the dedifferentiation of a resident vascular cell, needed to be ruled out.^5^

The risk factors for developing pulmonary intimal sarcoma are not yet specified due to the rarity of the disease. Individuals homozygous for the sequence variant of MTHFR677 and MTHFR 1298 genotypes, as in our patient, were found to have lower plasma folate levels and lower levels of DNA methylation. The genetic instability and the abnormal DNA methylation induced by these mutations could contribute to the genesis of this type of sarcoma.^6^

In conclusion, we reported a case of pulmonary artery intimal sarcoma that was misdiagnosed as pulmonary thromboembolism, and we also introduced a possible risk factor for its development.
